# Patient understanding and experience of non-invasive imaging diagnostic techniques and the liver patient pathway

**DOI:** 10.1186/s41687-021-00363-5

**Published:** 2021-09-10

**Authors:** Andy McKay, Carlos Pantoja, Richard Hall, Sarah Matthews, Phil Spalding, Rajarshi Banerjee

**Affiliations:** 1 Perspectum Ltd, Oxford, UK; 2 Liver4Life, (UK Registered Charity: 1152618), London, UK

**Keywords:** Liver disease, Qualitative research, Patient experience, Patient understanding, Non-invasive diagnostics, Patient support

## Abstract

**Background:**

Clinical and patient-reported outcomes are positively affected when efforts to increase patient understanding of underlying diseases and foster patient participation are part of care pathways. The prevalence of liver diseases is increasing globally, and successful communication of results from liver diagnostic tests will be important for physicians to ensure patient engagement and encourage adherence to lifestyle changes and therapy. Here, we aimed to explore the impact of non-invasive liver tests on patient experience and patient comprehension of liver disease in chronic liver disease diagnostic pathways typically managed with liver biopsies.

**Results:**

101 participants diagnosed with a range of liver disease aetiologies (90 patients, 11 caregivers) underwent a multiparametric magnetic resonance imaging (MRI) test. A subset of 33 participants was subjected to transient elastography (TE) with FibroScan® in addition to multiparametric MRI. MRI results were analysed using Liver*MultiScan™*. Participants received results on their liver-health status followed by a semi-structured interview to assess the scan procedure experience, comprehension of the results, and experiences of liver disease. A subset of participants (N = 5) was also engaged in the design, execution, and thematic analysis of the interview transcripts of the study. Analysis of semi-structured interviews revealed: (1) Presentation and discussion of the Liver*MultiScan* visual report by a physician was an effective contributor to better patient experience and increased comprehension of liver disease. (2) Patients demonstrated preference for non-invasive tests over biopsy for management of liver disease. (3) Patients reported positive experiences with the MRI test during the path for liver disease management.

**Conclusions:**

Patients presented with visual reports of liver test results developed increased understanding of liver disease care which may have contributed to an overall more positive experience. Patients reported that clinical information obtained through non-invasive methods and transmitted through visual reports contributed to clarity, understanding and overall increased satisfaction. We conclude that a shift toward non-invasive testing and visual reporting of clinical information (e.g. picture of liver with visual scale) when possible are likely to contribute to improved physician engagement with patients and lead to better outcomes in the management of chronic liver diseases.

**Plain English summary:**

Evidence suggests that patient experience and understanding can affect several aspects of clinical care and patient well-being. In this study, 101 patients and patient caregivers affected by liver diseases were recruited to determine how patient experiences of liver disease were affected with the introduction of non-invasive evaluation of the liver with an MRI or ultrasound-based elastography. All 101 participants received an MRI followed by a Liver*MultiScan* report. 33 participants received an additional FibroScan and report. Following the reports, participants were interviewed and asked to reflect on factors which affected their experience of the procedure and the understanding of their results. We focused on factors related to the layout of the standardised report and the delivery of its results. The interviews were transcribed and analysed for common themes and patterns. Patients and patient advocacy groups were involved in the design and conduct of the study, and analysis of the interview transcripts. Here, we report the perception of patients and patient caregivers on the quality of care and diagnostic experience.

*Trial registration* ClinicalTrials.gov identifier—NCT02877602.

**Supplementary Information:**

The online version contains supplementary material available at 10.1186/s41687-021-00363-5.

## Background

Chronic liver diseases represent a growing public health problem. Increased prevalence of liver diseases has been fuelled by increasing prevalence of obesity and related metabolic disorders, alcohol use, and viral hepatitis [1, 2]. Mortality rates from liver-related conditions have risen 400% in the UK since 1970 [3]. According to the Institute of Health Metrics and Evaluation [1], the contribution of liver cirrhosis to the proportion of total Disability Adjusted Life Years (DALYs) lost each year in the UK has more than doubled between 1990 and 2010, and continues to grow at an annualised rate of over 2%, amongst the fastest of any condition. Hepatocellular carcinoma (HCC), which shares risk factors with other liver diseases, has also been rising [4]. Consequently, liver disease and liver-related conditions are increasingly important for health systems. Key to reducing the burden of poor liver health is early diagnosis. Widespread implementation of early diagnosis will be necessary to aid prevention of advanced and disabling forms of liver conditions, and to minimize individual and societal costs [5].

Currently, liver biopsy is considered the ‘gold-standard’ for diagnosis of parenchymal liver diseases. However, the liver biopsy is an invasive diagnostic modality involving significant health risks and patient discomfort [6]. Liver biopsies are expensive procedures that generate significant healthcare costs secondary to inpatient procedures, in-hospital recovery time and biopsy-related complications. Finally, liver biopsy precision suffers from significant sampling and inter-observer variability in the interpretation of results [7–9]. Thus, there is a clear need for accurate and precise non-invasive liver diagnostic methods.

Non-invasive tests used to evaluate liver health can be divided in blood-based and imaging-based methods [10]. Blood-based tests are typically used to exclude advanced disease and/or trigger referral to more advanced testing or specialist liver clinics [11]. Imaging methods based on ultrasound (US) and magnetic resonance imaging (MRI) are becoming increasingly common and have the potential to replace the liver biopsy as the diagnostic gold-standard to monitor parenchymal liver disease at the population level [12, 13]. However, the effect of non-invasive imaging tests on patient-reported outcomes during liver disease clinical pathways remains relatively unexplored. The goal of the present study was to evaluate the impact on patient experience of the introduction of ultrasound-based TE with FibroScan or multiparametric MRI with Liver*MultiScan* on the diagnostic pathways of parenchymal liver diseases.

TE is a point of care technique that uses a special probe to measure the propagation of shearwaves through the liver to estimate a liver stiffness measure (LSM) that correlates with liver fibrosis [14]. Kan et al. reported that TE was preferred over biopsy by over 95% of respondents of a survey of patients that underwent TE at a Canadian hospital [15]. Importantly, patient preferences were independent of previous experience of a liver biopsy and a large fraction of patients were willing to self-pay for the TE test to avoid a liver biopsy. A qualitative study on the impact of TE on screening and diagnosis at the primary care level revealed that patients’ experiences were positive and that the explanation of the test results appeared to increase motivation for lifestyle changes to decrease the risk of liver disease [16].

Liver*MultiScan* is a multi-parametric MRI diagnostic tool that allows assessment of fat, iron and fibro-inflammation [13]. The Liver*MultiScan* cT1 score reflects fibro-inflammation and has demonstrated predictive value in non-alcoholic steatohepatitis (NASH) disease progression, autoimmune hepatitis disease activity, and ability to monitor responses to treatment in patients with hepatitis C [17–20]. Recently, the Liver*MultiScan* cT1 score has been recommended for the evaluation of patients at risk of non-alcoholic fatty liver disease (NAFLD) as part of a pathway that includes multiparametric MRI [21]. In addition to addressing the patient discomfort occasioned by the liver biopsy, cost-analysis has demonstrated that non-invasive diagnostic modalities, including Liver*MultiScan*, are cost-effective in comparison to the standard diagnostic pathways for liver diseases [22]. The Liver*MultiScan* report includes images of the liver with an embedded red-green colour scale that depicts variation in cT1 values across the liver parenchyma. cT1 values under the upper limit of normal are depicted toward green and cT1 values above the upper limit of normal, which correlate with fibroinflammation, toward the red end of the colour scale [23]. Currently, there are no published studies evaluating the impact on patient-reported outcomes of including Liver*MultiScan* in the pathway to diagnose and screen liver diseases.

To contribute to optimal clinical outcomes, diagnostic tests must not only demonstrate clinical value and cost-effectiveness, but also address patients’ needs. A recent survey of patients with liver disease conducted by the British Liver Trust revealed that 59% of people felt the need for more information during the diagnostic process and 90% sought additional information online after the clinic appointment [24]. Similarly, Cook et al. surveyed patients with NASH in the US and the UK and reported poor understanding of the disease by patients and inadequate physician support [25]. Collectively, these studies suggest a failure to successfully integrate patients during the diagnostic process, resulting in poor understanding of the nature of tests being administered and/or the nature of the diseases being diagnosed. This highlights the need for diagnostic approaches that provide not only clinical utility, but also information that is understood by patients and can be used to promote patient education of disease progression and empowerment toward self-management [26]. Here, we sought to investigate the impact of Liver*MultiScan* and TE on patients’ experiences using a qualitative, survey-based approach that included investigating if information presented by way of a test with a visual component could affect patient understanding.

## Methods

### Study design

The study design and questionnaire were first conceived by one of the authors and were finalized in conjunction with five individuals with experience of liver disease (3 patients and 2 caregivers) members of a liver disease charity. Design participants took part in a facilitated meeting to review the study protocol, the participant information leaflet and a standardised questionnaire. Including a quantitative questionnaire allowed a self-reported value of understanding of liver health before and after the liver scans. This was measured by patients selecting a value between 1 and 10. Mean scores were calculated for those having the Liver*MultiScan* test, Liver*MultiScan* and the FibroScan tests, and the average for all participants. Participants were encouraged to give recommendations on changes to be made to items such as the format of reporting of the MRI scans after receiving their own reports, as opposed to dummy reports. Participants’ suggestions were implemented into the final study design.

**Fig. 1:**
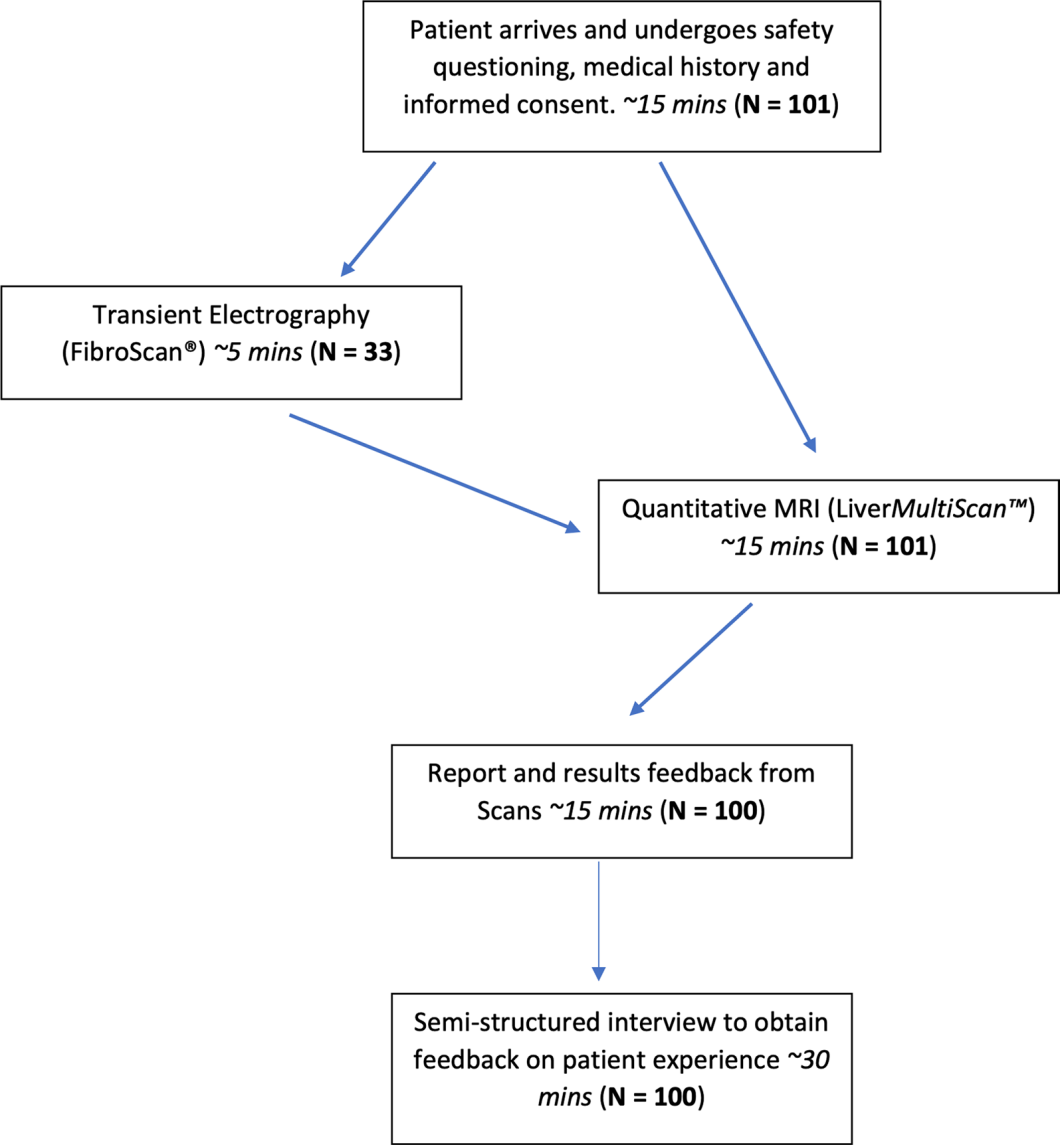
Flow-chart of patient care pathway on day of study

All participants attended one visit lasting no more than two hours. During the visit, participants first underwent an MRI scan, had their MRI report presented by a healthcare professional, and then engaged in 30-min semi-structured qualitative interview as shown in Fig. 1. A subset of 33 participants also underwent a FibroScan test, delivered by a trained consultant physician, either before or after the MRI in a pseudorandomised order.

### Magnetic resonance imaging

MRI data were acquired on a 3 T Siemens Tim Trio scanner and analysed with Liver*MultiScan* (Liver*MultiScan*, Perspectum Diagnostics Ltd, Oxford, UK) software. Scan time was < 10 min, as previously described [18]. Briefly, transverse abdominal T1 and T2* maps were acquired for estimation of extracellular fluid and liver iron, respectively. This was used to generate the iron-corrected T1 (cT1) for a composite measure of liver inflammation and fibrosis. Liver fat was calculated from the proton density fat fraction (PDFF). Data was then analysed to generate the Liver*MultiScan* report (Additional file 1).

### Transient elastography (FibroScan®)

Transient elastography (TE) measurements were determined with a FibroScan model FS502 with ‘M’ or ‘L’ probes. The TE test is performed under 5 min, with an average of 10 valid measurements being used to obtain a liver stiffness score (kPa) which is correlated with fibrosis staging [27]. The score can be compared to the FibroScan scoring card (Additional file 2), which can indicate the likely fibrosis staging of the scanned individual (F0–F4).

### Semi-structured interview and questionnaire

Healthcare professionals discussed Liver*MultiScan* and FibroScan test results with the use of visual reports in a one-on-one setting. Immediately after, a study investigator conducted a 30-min semi-structured qualitative interview. First, each participant was given a few minutes to examine the Perspectum Liver*MultiScan* Questionnaire (Additional file 3). Next, the interview was conducted using open-ended questions in a semi-structured fashion to probe: (1) experience of the scan(s), (2) understanding of the report, and (3) ways to improve delivery of both the scan experience and scan report document. Participants filled in a questionnaire, designed in conjunction with patient advocacy groups and patients, before and after their scans and feedback. The questionnaire collected information about participant background, information delivery preferences and how understanding of liver health changed after receiving scan reports (Additional file 3).

### Analysis

Interviews were transcribed verbatim before thematic analysis. Thematic analysis followed a grounded theory approach, where theory is itself generated from the qualitative data of the interviews. All transcripts were annotated and coded for subject matter. The transcripts were then analysed a second time to identify portions of text which related to identified codes and to determine whether the selected text was positive, negative, or neutral on the subject matter in question. Quotations were organised by code and analysed to determine links between different subject codes. Coded quotations were organised into groups linked conceptually from which specific themes (summarised in this paper) emerged. A three-person patient panel reviewed the transcripts and analysis at two time points to validate the analysis.

### Researcher reflexivity

All the authors of this study have had personal experience of liver disease or direct experience of the journey of patients diagnosed with liver disease as physicians. The author who conducted the interviews and coded them had been evaluated with the Liver*MultiScan* test prior to the study conception and their personal experience with the technology was one of the contributing factors that spurred the interest in evaluating the effect on imaging non-invasive technologies on the experiences of patients being evaluated for liver disease. Prior to this experience this author had had no contact with Liver Disease Charities or other types of patient groups. In addition, none of the interviewers had any direct relationship with the participants of the study prior to the interview.

## Results

### Participant demographics

**Fig. 2:**
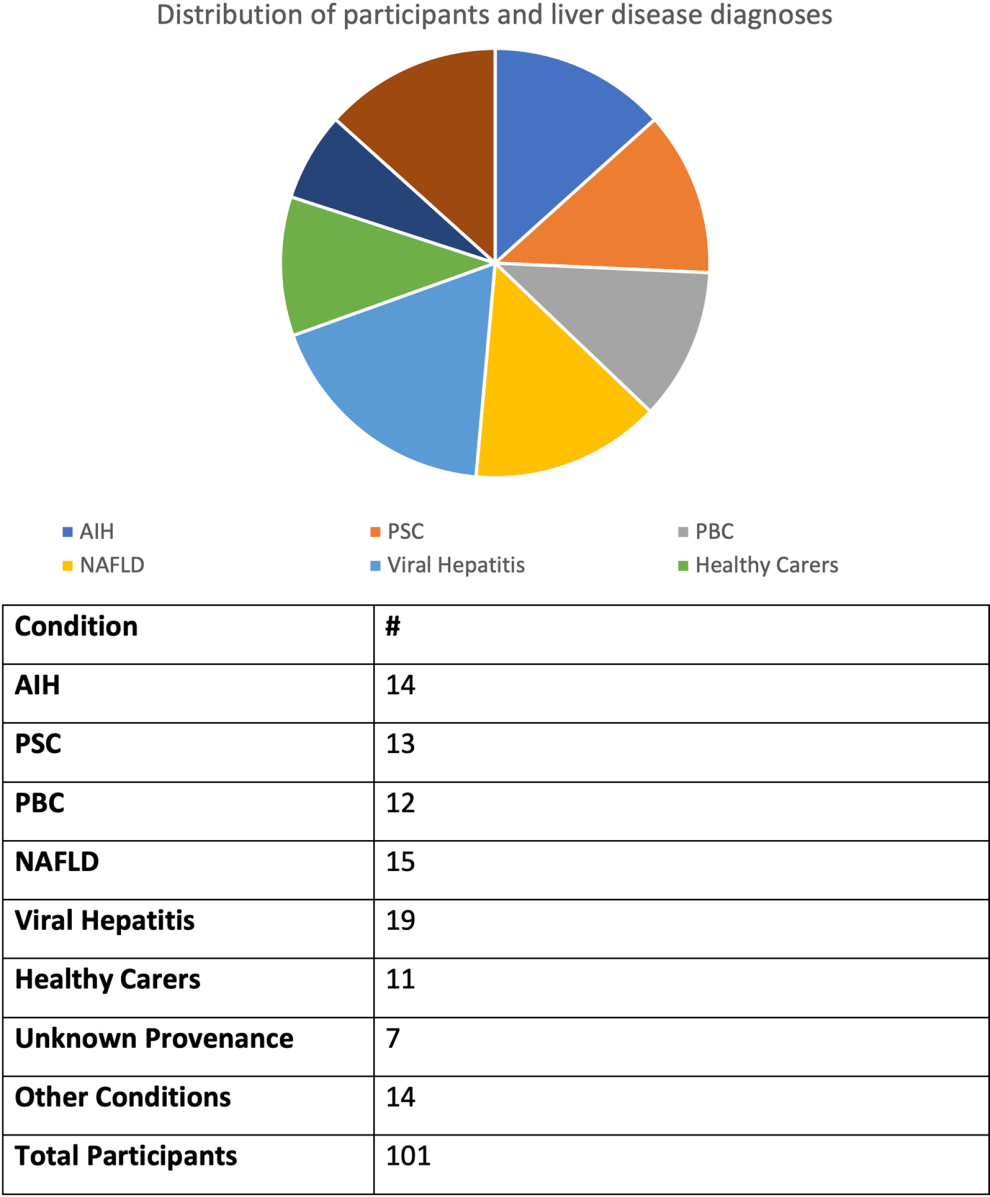
Numerical distribution of participants and liver disease diagnoses. Cohort of 101 individuals with diagnoses including overlap syndromes—i.e. AIH/PBC overlap syndrome (Sum of all diagnoses adds to more than 101). Other conditions include liver cancers, rodular regnerative hyperplasia, alcoholic diver disease, cystic liver disease and haemochromatosis. Liver disease diagnoses were self-reported

A total of 101 participants (62 females, 39 males; aged 20–79, mean 51 years) provided informed consent to participate in this study in accordance with ethical approval from Oxford-C South Central Research Ethics Committee (Ref: 15/SC/0615). The study population consisted of 90 participants with previously diagnosed liver disease and 11 caregivers. Participants were recruited from liver support groups and charities, Perspectum’s social media and online platforms, and direct invitation from other participants. The exclusion criteria were contraindication to MRI scans and age < 16 years old. Patients not meeting the exclusion criteria were enrolled until 100 participants were accounted for. Diagnosis was self-reported, and the frequency distribution of diagnoses reflected the convenience sampling strategy that was utilized. Notably, the final study population contained no participants that self-reported alcohol-related liver disease, demonstrating undersampling of the population and/or possible avoidance of self-reporting alcohol-related liver disease. Distribution of liver disease diagnoses is shown in Fig. 2.

### Identified themes

Five core themes were identified:

- MRI procedure
- Non-invasive versus invasive diagnostics
- Understanding presented information
- Delivering information: access is assurance
- Patient support and care post-diagnosis

### The MRI procedure

Perfectly comfortable, unexpectedly in a way

Example quotations can be seen at Table 1.

**Table 1:** MRI procedure example quotations

“It all felt quite straight forward and comfortable” Mark, 56M with AIH
“There is one thing I feel I should bring up…. I have COPD and I know that some of peers also have problems breathing. There’s a lot of breathing involved when you are inside the machine… if I had known I would have had this (inhaler) before I went in” Charles, 58M with Hepatitis C
“Because you can’t put them on yourself, you can’t do them up. You feel like you are walking around in corridors, in your nightdress.” Jessica, 35F with AIH
“It was very straight forward. Far more than I was expecting. It’s non-invasive. It is noisy, the headphones keep that out.” Nick, 34M with NAFLD
“There is no pain involved at all. You are just laid down. It’s easy, and that’s it. It’s quite comfortable. You are in a good position and it’s relaxing” Taylor, 48F with PBC
“I am very claustrophobic, so it’s never going to be an entirely comfortable experience, but it was okay. It helped a lot that the assistant kept talking to me, as she knew I was fairly anxious. That helped a lot.” Shirley, 55F with AIH

The majority of individuals felt that the MRI Liver*MultiScan* was tolerable, with a few individuals even stating enjoyment or relaxation from undergoing the procedure. Participants generally felt the information they received through the Liver*MultiScan* and FibroScan reports was more than what they initially expected and was sufficient in quantity and quality. Most participants thought it useful to have information prior to the scan and felt that the option of a paper or electronically delivered leaflet was a good way to do this.

In terms of preparation for the MRI scan, 4 h of fasting was not considered a major impediment or sacrifice, particularly if good results were obtained. Occasionally, the preparation for the scan generated anxiety due to potential conflicts with daily drug regimen, but after speaking with a member of the research team, solutions and alleviation of any major stress were possible. Participants appreciated the ability to wear their own clothes into the scanner, as opposed to a hospital gown which many identified as a time waster, a drain on their dignity during the procedure and a contributor to anxiety by enacting an overtly clinical atmosphere to the scan. Several participants commented positively on the use of a pillow by the radiographers, stating that a flatbed without such support is what they had been used to in the past.

Most patients found the scan procedure harmless. The relatively tight space was expected, and those who were mildly claustrophobic (minority) found ways to cope with this by closing their eyes or requesting a blindfold. The larger space posed more of an issue in larger participants, usually the cases of NAFLD or NASH, with a bore size of 60 cm being used. On average, the sound during the scan was more unexpected and sometimes caused discomfort. Temperature inside the scanner also presented as an issue of discussion, though primarily due to the protocol causing warming inside the scanner. Many also stated how much an occasional verbal message from the radiographer during the scan helped their experience, emphasising the importance of human interaction as a moderator for patient anxiety.

Breath holds are essential to obtain good images within the Liver*MultiScan* protocol. Most participants did not have issues with the breath holds, and successfully followed instructions. Participants with co-morbidities that affected breathing were more likely to report difficulty with the MRI exam. Others said they would have liked a more practical demonstration of the breath holds prior to the scan as opposed to verbal description only. The negative experiences and perceptions mentioned above should remain in their context, as most individuals had very little problem with the MRI scan. Individuals with past experience of the scan stated that their anxieties and worries about MRI scans also generally disappeared after their first MRI scan.

### Non-Invasive and Invasive Diagnostics: Liver*MultiScan*, Biopsy and FibroScan

I don’t want a biopsy.

Example quotations can be seen at Table 2.

**Table 2:** Non-invasive and invasive diagnostics: Liver*MultiScan*, Biopsy and FibroScan example quotations

“Biopsy was very stressful and very painful. (The MRI) was a walk in the park in comparison to that.” Mary, 51F with Haemochromatosis
“It took me a long time to get over that one. At the time—it was okay. It was the recovery stage after that. It felt exactly like you had been stabbed, which basically I suppose you have been.” Clive, 69M with AIH
“Because I had one recently that did feel quite stressful. This one seemed—I knew what to expect. So, I didn’t feel stressful and I didn’t feel weird afterwards like the last one.” Anna, 35F with AIH
“For me, I didn’t feel anything. I just had a bit of a chill out for ten minutes. I laid back and relaxed.” Alan, 41M with Hepatitis C
“MRI’s are just a short thing out of your day. You sit, you lie down, relax or at least try to relax. Have some loud noises, feel a bit warm, you get out and you get diagnosed and that is sort of it. As procedures go—it’s very casual.” Rowan, 18M with NAFLD
“At the start, it was uncomfortable, it was a tight space. But once you were inside, it was comfortable enough. I think it is just that original going into the tunnel.” Marcus, 25M with Unidentified Condition
“Well, the fibroscan is really quite benign really I think actually. Fibroscan is so short relatively” Charles, 58M with Hepatitis C
“Because it was non-invasive. It doesn’t cause me any problems. It’s quick, it doesn’t affect anything. Whereas the alternative, a liver biopsy would be completely the opposite.” Julie, 43F with NAFLD
“I had two biopsies. I had one in 2011 and one in 2014. It’s excruciatingly painful… And then you go back home and this pain comes there for a number of days to heal up. Then that alone itself—the second time I felt like I was going to have a panic attack… The drama that goes with the liver biopsy. They are preparing you like an operation—it’s traumatic. You know, they make you feel as if they are going to chop you up. I wouldn’t want to go through any liver biopsy again.” Victor, 36M with Hepatitis B

Nearly all the comparative procedural discussion was between multiparametric MRI with Liver*MultiScan*, TE with FibroScan and liver biopsy. Participants were mixed in their experience; with some having undergone all three, and others having one of the three for the first time as part of the study. The experiences related to the liver biopsy displayed a common theme. Participants let it be known that they found biopsy very uncomfortable physically, and subsequently a great cause of psychological stress. The idea of a repeat biopsy being a cause of additional stress was a recurrent theme for participants, and one they wished to avoid. Indeed, many participants told stories and anecdotes of how ill they felt after a biopsy procedure. Participants seemed to prefer TE and MRI because they were non-invasive, short in duration and with results being delivered relatively fast. A minority of patients mentioned the discomfort caused by the touching and pulsing elements of the probe used for the TE test. However, patients generally spoke warmly of modern technologies which would help them understand their liver better, and potentially with more ease. Several patients discussed other trials and studies of technologies they have been involved in with warmth.

Whilst patients did value good, clear and personalised results as a priority, a desire for non-invasive diagnostics was strong. An overall preference was given to a diagnostic test that could provide a non-invasive experience and solid results.

### Reporting—understanding presented information

When you actually look at the pictures, you can tell the story. That is what it feels like.

Example quotations can be seen at Table 3.

**Table 3:** Reporting—understanding presented information

“I think it helps you understand things. Often, you get blood results with a number or an MRI, they’ll just write a line…Whereas when you see an image, it’s easier to remember and understand.” Natalie, 35F with Autoimmune Hepatitis
“I prefer the visual aid. The text is okay, but when you get a letter back from your consultant and it is full of the technical, the first thing you do is go on google… You are less worried (with the picture) than a report saying you have some steatosis” Matt, 45M with NAFLD
“Seeing the liver and what state it is in, rather than just being told you have F3 or F4. You know, you don’t understand… you don’t actually see the bits which are damaged.” Jo, 48F with Hepatitis C
“I feel I can close the book on my liver. Does that make sense? Even though I had Hep C 6/7 years ago now, I’ve always wondered how much damage is there, as they only take a minute part of the liver when they have the biopsy. I always wondered, so I’ve come and I’ve got the confirmation I was after. I didn’t know it would be so good, but I’m very happy it is. So for me, having a history of (Hepatitis C), this have given me a lot of security to know I am in good shape” Michael, 41M with Hepatitis C
“The colourfulness makes a massive difference. The CT scan, MRI scans. All these scans. They are in black and white. The (radiologist) just points to your liver, this it that etc., but it doesn’t make sense to a layman like me.” Victor, 36M with Hepatitis B
“When I sat down, the first thing I wanted to see what my number. I wanted to see my score. I wish that that would have been bigger, because I had to keep asking.” Simone, 52F with Primary Sclerosing Cholangitis
“it shows you exactly where the diseased parts of the liver are which is what I haven’t ever known in the past” Lorely, 58F with Nodular Regenerative Hyperplasia
“The visual clarity is just stunning isn’t it, and so easy to understand. Because you haven’t done anything fancy, you’ve just used red, amber, green. So I can see at a glance” Lynne, 55F with Autoimmune Hepatitis
“As I said to you earlier, it’s an encouragement to keep going with the lifestyle I’ve been leading for the last few years. That’s fantastic.” James, 60M with Hepatitis C

High-quality visual reports of tests and discussions with a healthcare professional during the diagnostic experience appeared to improve participant experience and comprehension of liver disease. Participants felt that the MRI scan delivered them clear and understandable information. Central to this was the pictorial and colour elements of the Liver*MultiScan* report. The Liver*MultiScan* report contains a prominent colour-scaled image, with relevant statistics above this image. The colour scale is to the ‘cT1’ reading, which itself is a measure of inflammation and fibrosis—red representing high cT1, and green/blue representing low scoring. Participants acknowledged the understanding provided by the pictorial element of the scan report because they could clearly see and assess the entire liver. This was particularly so for those with patchy disease, for whom such assessment is a significant advantage over other methods such as biopsy which assess only a restricted volume of the liver. The use of imagery that spanned the whole liver also increased confidence in the test for many, reassuring participants that they had been fully assessed.

Most prominent amongst participants responses to the visual aspect of the report was the colouring of the picture. Primarily mentioned was its explanatory power which was reassuring to participants. Many stated that the colour helped them understand their condition far better than verbal or numerical explanations, telling an accessible story of the health of their liver.

Participants with liver disease in recovery, or who had been subjected to a liver transplant were also reassured by the visual elements of the report with a picture of the whole liver. These participants often reported anxiety and concerns about present liver health or if they had really recovered. Several participants reported that they felt this type of report could help with a ‘closing of the book’ and a moving on moment for the psyche of an ill person. Liver-transplant recipients reported that doubts about the condition of their new liver could be resolved. The colouring, a universal messenger, was the key element allowing patients to grasp a general message about their state. To supplement the visual report, participants also valued the numerical aspect of the report. Generally, participants felt that the numbers were the core of what ‘you need to know’, to aid in comprehension of the full picture of liver disease severity.

Most participants understood the numerical aspect of the report. However, a few struggled with interpretation. Participants who struggled with interpretations struggled due to signage, normal ranges, and nomenclature. Participants most likely struggled with nomenclature because new and unique acronyms were utilized in the report without references. Difficulty with colour differentiation occurred in patients with intermediate liver conditions. For example, vessels on the visual report were a yellow colour. Physician-guided discussion of normal versus abnormal patterns in the Liver*MultiScan* report was critical for full comprehension of the report by participants.

### Delivering information: access is assurance

It’s my body and I’m doing whatever I do to it – so I want to be a partner. Whatever you know, I want to know as well

Example quotations can be seen at Table 4.

**Table 4:** Delivering information: access is assurance

Delivering Information: Access is Assurance Example Quotations
“I don’t know much about the statistics. That is gibberish to me.” Alice, 40F with AIH
“The consistency of seeing the same people as well when whenever you go see a consultant, you see somebody different every time. Even though it’s the same consultant, the people that you see are different each time. And some of them will actually talk and answer questions and others, you are in and out in 2 min, and they don’t look up from the computer. Jenny, 58F with NAFLD
“It’s my body and I’m doing whatever I do to it—so I want to be a partner. Whatever you know, I want to know as well” Jamie, 58F with Unknown Condition
“It’s very difficult actually—because you have to request them all the time. And now our surgery has started charging… 47p a sheet… so every time you go and have a blood you have to pay 5 or 6 quid.” Christopher, 69M with AIH
"The first time you will need someone to go through it with you very clearly. To spend time, and let you ask questions so that the first time it is contextualised and you learn how to read it and then from then on you know what to do. It’s almost like you need a little bit of training.” Alex, 51M with PSC
“What was bad was the consultant who I saw, who seemed to have zero emotional intelligence. He was a clever idiot is how I put it.” Matt, 62M with Hepatitis C
“They don’t show you nothing. Normally when they do it, you don’t get to see the screen. Today, I saw the screen and when they do it in the hospital, it’s normally turned away from you. So you can’t see anything” Shirley, 63F with NAFLD
“I love it and wonder why we don’t have them. I mean our GPs don’t even get an image of it either. My spinal MRI’s, instead of getting an image of it, they get a letter explaining what the MRI does.” Alex, 51M with PSC
“I think the image is good, I think it’s reassuring, it’s nice to have, but if you have much more detail than that, then there is more scope to worry and some patients I know would grasp on to every tiny piece of information and one amazing detail about all the… and I’m not like that, but some might be.” Sarah, 48F with PBC
“I just feel slightly better if I have the chance to get as much information as possible” Sophia, 68F with Unknown condition
“I think the problem is when you have things wrong with your internal organs, you can’t bloody see anything… You don’t know what is happening. To see an image of your insides, even if it was awful, you feel at least you have the knowledge and have some power. It’s empowering.” Alex, 51M with PSC
“There’s more in there than I could explain to you, because since the transplant, I’ve had lots of thoughts from time to time. I used to in the old days, couldn’t afford a good car, so I had a scrap car and I always had to keep going to the scrapyard to get spare parts, and some of them were alright, and some of them—you would change the engine and two months later, you did again. I had no idea personally of what condition the liver was in except obviously it was good enough, but, a question I’ve asked and never had a reply was what age the person who owned this liver. You know, was it somebody 35 or was it somebody 75? Because then you think, how long is that liver going to last? You know, but seeing that now tells me it’s a healthy liver, so that has gone into insignificance. That question has gone from my mind now, because it doesn’t matter. That is what matters.” Derek, 63M with previous Liver Transplant (NAFLD)

Understandable and careful information delivery by a doctor or health professional was considered essential to assuring patients of the quality and validity of their results. Participants wanted to know that their information was delivered with expert approval as this may increase confidence in results and lessens stress—although many felt that current standards of delivery were not consistent and often did not aid them in their understanding. Adequate delivery of test results also offers the chance to ask questions and resolve doubts important to one’s understanding of a personal medical condition—a very important aspect of a health interaction for the patient. Where a scan showed a healthy outcome, there was less desire expressed for a face-to-face consultant, with many saying in this case they would be content with just a written letter or the test results being communicated by other health professionals.

The aspects identified as central for a good rapport and understanding of test results were: sensitive and patient-oriented discussion of results with the use of visual props. Many patients mentioned feeling misunderstood and having trouble understanding test results in the past because of poor exchange of information with the attending physician or healthcare professional. Several participants informed that doctors did not communicate on layman’s terms—delivering information without properly explaining many terms. Participants also thought that information was in some way concealed from patients by what was described as the ‘behind the desk’ culture of reporting results; with images/results not shown from behind the screen and leaving the impression that not all relevant information had been covered. Many felt a general opacity in terms of delivery of their own information. Many participants also explicitly stated that having access to a visual prop on a screen allowed them to see that all was being covered, and prompted them to ask questions which alleviated stress. Use of comparative examples was also praised. This more open form of delivery was noted as empowering by patients more than once, allowing them to form a more partnership-style relationship with their doctor.

Participants showed a strong desire for a paper or electronic copy of their report. Identified reasons for this included keeping records of their own for self-tracking, but also acknowledgement that they and the population in general are much more mobile and thus there is an advantage to having quick access to reports if a change of doctor is at hand, since information is often lost in the transfer process.

Most participants understood that they could not possibly understand test results at the level of a medically trained person, but they did not wish to miss out on information, and generally felt that where information was present, it should be made available. Many went as far as to say that they could not receive too much information. This was particularly present in participants with higher measures of fibro-inflammation. The general theme that discussion of test results with emotional and sensitive intelligence, coupled with an open form was the method preferred by participants, demonstrating good communications skills being an essential skill for a healthcare provider.

### Support, care and post-diagnosis

The empathy comes from a human.

Example quotations can be seen at Table 5.

**Table 5:** Support, care and post-diagnosis

“I walked in there and there were two guys, similar age to me who’d had a similar experience in terms of the longevity of the infection and they had both been through the treatment and been successfully treated and were feeling much better. So it was a real—just to hear someone else’s story. For me, it was vital.” James, 60M with Hepatitis C
“It is good that you can have a moan and people understand that moan, and people are going through the same thing as you understand it. Whereas when you are talking to general public, you don’t like to keep on—you feel like a whiner kind of thing. When people ask ‘how are you?’, I often say I’m fine when I am not—because they don’t want to hear it. Whereas on the support group everyone is sharing their experiences and you feel like you can do that.”45 with PBC
“The thing is any knowledge I have about the condition I have has been provided through the support network that we have. Doctors have never sat down with me and said this means this and this means this. If I have an understanding, it’s one I acquired myself from using the support mechanisms that are out there through voluntary organisations that support us.” Jean, 43F with PSC

A general emerging theme was that many patients did not feel supported during the healthcare journey. Patients reported insufficient condition-related guidance and emotional support, which lead patients to express that liver conditions might lack solutions/treatments and are poorly understood. Participants with a diagnosis of NAFLD reported these feelings more often than participants in the other diagnostic categories, indicating that decreased patient support could be a characteristic specific to the NAFLD patient journey. In fact, many participants with a diagnosis of NAFLD greatly valued support groups. Support groups seemed more concentrated within certain conditions than in others, with some conditions having more empowered and active patient groups. Many participants who were engaged in groups saw them as an essential part of their condition management because it facilitated interactions with other individuals experiencing similar diagnoses. This was reflected in some of the stories and anecdotes told by participants, many of which focused on a theme of being ‘left behind’ by healthier friends and family members who did not understand the full impact of a liver condition on the participant’s life. Other participants struggled to find sufficient support, and stated that the personal engagement offered by support groups could be highly valuable. Both meetings, and ward walk arounds by former or current patients were praised.

Support of online-based patient groups was also mentioned. This was primarily for the ability to ask questions and get quick responses. Others were more reserved in relation to online groupings, citing uncertainty in relation to quality of information given, and difficulty interacting within groups that often have many different personalities with discussions between members moving fast.

Touching on the quality of information given out on groups, many participants explicitly stated they often did their own research through internet search engines or online support groups. Many understood that this would not necessarily lead to reliable information. Support groups were seen as guardians of more reliable information, and could be a potential mechanism for information dissemination.

### Quantitative questionnaire

Self-reported understanding of liver health increased significantly (1% significance level, paired Student’s t-test). For all participants, this increased from 6.20 to 9.31 (+ 3.11). For those who just received Liver*MultiScan*, there was an increase from 6.28 to 9.22 (+ 2.94), and for those who received both went from 6.03 to 9.45 (+ 3.42).

In addition to participants rating the understanding of their own liver condition, the 11 caregivers were asked to rate understanding of their associated participant’s condition. This was also on a scale of 1–10 with an average score of 8.04. Understanding of sufferers of each condition varied. The average understanding for the five conditions with an N > 10 were calculated; primary sclerosing cholangitis (PSC), primary biliary cirrhosis (PBC), auto-immune hepatitis (AIH), NAFLD, hepatitis C. Hepatitis C participants claimed the highest understanding at 8.17, with NAFLD participants lowest with 6.73. AIH participants were 8.08, PSC 7.38 and PBC 7.58.

The subsample of participants (N = 33) who received both multiparametric MRI and transient elastography were asked to pick a preference for tolerance, usefulness and inclusion in the treatment pathway. Opinion was split on tolerability of the tests with 24% each saying they preferred either multiparametric MRI or transient elastography, but 52% stating no preference. However, 88% found multiparametric MRI more useful, and 91% said they would prefer it over transient elastography.

## Discussion

The present study shows increased understanding of liver disease by patients and improved patient experience with utilization of TE with FibroScan and multiparametric MRI with Liver*MultiScan* in the diagnostic pathway of liver diseases. In addition, patients reported increased satisfaction and understanding of their condition with the utilization of visual reports as part of a patient-focused clinical experience. Patient experience is an important factor to investigate within clinical pathways [28], and has been linked to several important indicators including satisfaction [29, 30], loyalty [30], clinical effectiveness [29, 31], compliance [32] and outcomes [33]. Furthermore, there can be discrepancy between physician assessment of patient experience, and self-reported patient experience [34]. Finally, clear methodology and presentation of results obtained through research focused on the patient experience is important to ensure that all stakeholders are active participants in a study and to increase the likelihood of impact in real world practices [35]. In the present study we applied the principles of patient and public involvement in research (PPI) [36] to investigate non-invasive diagnostics for liver disease. Our results indicate that inclusion of Liver*MultiScan* and FibroScan in the clinical pathways to manage liver disease might improve patient experience in real-world settings and aid the patient physician relationship toward promoting and maintaining lifestyle changes through empowerment.

Since test scans were performed within a research environment, fewer scheduling constraints occurred compared to real-world clinical settings. Thus, the study setting might have introduced a bias on patient experience toward positive impressions that might not reflect patient experiences during real-world clinical visits. We mitigated the risk of study setting-bias by setting a schedule which reflected real-world clinical practice. During each study day, 8 to 10 participants were scanned from 9 am to 4.30 pm. This interval included a lunch break. We believe this approach enabled a more realistic experience during test scans.

The study population was likely shaped by self-selection bias related to potential claustrophobia risk in some participants during MRI scanning. Eligible participants with propensity for claustrophobia might be discouraged to apply for participation. This bias could not be addressed because good clinical practice guidelines demand that participants with propensity to claustrophobia be excluded from MRI testing in a study setting.

Our study is affected by a design-bias intrinsic to qualitative approaches using interview-based analytic tools. To minimize this effect, we utilized a semi-structured approach to interviews. Question structures were explicitly neutral and participants were encouraged to be open. We expect that the large population size and variety of social and ethnic backgrounds of our study cohort reflect the real-world populations affected by liver diseases. In addition, volunteer participation in research studies might indicate increased “patient activation, which has been previously associated with increased satisfaction during healthcare utilization. Finally, interviews were reviewed by a group of patient group experts at halfway point and at the end of the study. This review concentrated on whether the analysis completed on the interviews was a fair representation of the feedback given, taking into account the interview structure.

The interviews conducted in the present study reveal that patients diagnosed with liver disease are interested and ready to discuss a wide range of aspects of liver disease care. Participants discussed many practical issues related to the diagnostic procedures and how information is transmitted to them. Participants revealed several emotional aspects and problems faced during the experience of liver disease. Patient participants helped set the structure to uncover important themes and clarify the themes during the post-interview analysis. Patient group involvement in dissemination of advertisement for the study also significantly eased recruitment, a factor which can hold back many projects. Notably, one of the authors of the study had a diagnosis of liver disease initiated through a Liver*MultiScan* test. Although this introduces a potential bias in our study it also provided a direct experience of the liver disease diagnostic journey that informed the study design and execution, but that is often absent when researchers without direct experience of the patient journey design and conduct studies to evaluate patient-reported outcomes.

Upon consideration of the interviews, three layers of patient experience were apparent: information, understanding of that information, and support of information. Through a thematic analysis these were sub-divided in five specific categories (MRI procedure, non-invasive vs invasive, understanding information, delivering information, patient support). A uniting desire of all patients, and one which was consistently present, was the desire for information access. Participants demonstrated that the current information offerings during typical clinical encounters were often inconsistent. Given that better understanding can lead to better outcomes, and information exchange between patients and physicians, patient comprehension should be prioritized. In this study, patients preferred visual aids and open reporting styles to communicate liver status and progress. Better understanding of an improving state has potential ramifications for the mental health of an ill or previously ill patient. Several interviewees who identified as recovered for several years spoke of their unease and the discomfort of misunderstanding their recovery and welcomed methods to improve understanding of recovery.

Whilst patients desired access to their results as a priority, they also greatly valued informative discussions of their results with their physician. Increased understanding of the disease through adequate presentation and discussion of test results was described as being helpful as part of the experience of feeling empowered to cope with a liver condition and/or toward recovery. There was preference for an open style of information presentation, which encouraged patients to ask questions. Visual reports were preferred by patients and seemed to contribute to better understanding. Easy and convenient access to test results, during and after the consultation, was thought to contribute to patient empowerment.

The preference for the non-invasive tests and aversion to invasive biopsy is not surprising. Patients who expressed aversion to confined spaces, or repeated pulsations of the TE probe also preferred non-invasive tests. It is also possible that participants showed reduced baseline stress in a research context relative to patients referred to MRI scans because of a clinical referral. This result suggest that patients are likely to benefit from clinical settings that are willing to adopt non-invasive technologies to evaluate liver health. Participants expressed the desire to participate in trials to develop and test new technologies. It is worth noting that the quality of information received was perceived as a primary concern, but if the necessary information can be obtained non-invasively, a strong preference for the non-invasive test was identified.

Finally, support groups and other mechanisms of community involvement should be considered. Recruitment for the study was done in conjunction with support groups and charities, but some individuals were recruited individually, via direct advertisement or targeted social media advertising. Generally, participants felt that support group structures eased their experience. Several participants received care in settings where groups were explicitly weaved into the care experience by providers. Hepatitis C and autoimmune liver conditions have established patient support groups whereas fatty liver diseases have fewer post-diagnosis support groups. Patients with NAFLD reported less understanding of their conditions on the 1–10 scale than patients with viral and autoimmune liver conditions. Participants integrated into patient groups were more positive about past experiences and felt that with support structures they were better able to understand the context of their personal state in reference to others, and cope with their condition. Many participants attributed the understanding of their condition primarily due to interactions with support groups, as opposed to healthcare structures. Support structures were reported to help with understanding by providing access to trusted information on patients’ conditions, and results interpretation. It may be that the lesser understanding amongst NAFLD patients’ may be due to poor identification with the disease. Through all this, a desire for the human touch prevails. Good delivery in a sympathetic and appropriate way, support structures which enable access to real humans with lived experience of the relevant condition. Patients often stated that in their experience of healthcare systems, they often felt alone, or pushed through as if a number.

## Conclusions

The present study sought to document the experiences and perceptions of non-invasive diagnostic pathways by liver disease patients. We have identified that non-invasive tests are highly preferred over liver biopsies, and that the use of visual aids greatly contributed to understanding and might impact patient reported outcomes during management of liver diseases. Importantly, NAFLD patients seemed to have less understanding of their condition and likely have access to fewer organized patient-focused support groups in comparison to other chronic liver disease conditions. This finding has important public health implications at a time when it is estimated that approximately 30% of the Western population have fatty liver disease and NASH is becoming the main cause of liver transplantation.

Our study reinforces the need to rethink the balancing demands for efficient treatment and optimal patient experience. Improvement in our current structures of healthcare management for liver disease patients will require structural thought and improved design of not only patient-centered studies such as this one, see GRIPP2 short-form (Additional file 4), but also incorporation of patient-initiated research priorities and patient reported outcomes into conventional pharmacology and interventional studies. The evidence from prior studies as well as the stories of patients with this study demonstrate the value of patient-centered outcomes as integral to the maximisation of utility within healthcare systems. We hope the insights provided here will inform future research and serve to improve patient experiences and care within liver diseases.

## Supplementary Information

**Additional file 1.** Representative Liver*MultiScan* Report.

**Additional file 2.** Representative Report of Echosens FibroScan® Report Card

**Additional file 3.** Patient Group Designed Questionnaire.

**Additional file 4.** GRIPP2 short form.

## Data Availability

All original data and interviews generated by this study are available upon reasonable request.

## Abbreviations

AIH: Auto-immune hepatitis
DALYs: Disability adjusted life years
HCC: Hepatocellular carcinoma
cT1: Iron-corrected T1
MRI: Magnetic resonance imaging
NAFLD: Non-alcoholic fatty liver disease
NASH: Non-alcoholic steatohepatitis
PBC: Primary biliary cirrhosis
PSC: Primary sclerosing cholangitis
PDFF: Proton density fat fraction
PPI: Public involvement in research
TE: Transient elastography
