# Supplementary material for: Patient understanding and experience of non-invasive imaging diagnostic techniques and the liver patient pathway
Source: J Patient Rep Outcomes. 2021 Sep 10;5:89. doi: 10.1186/s41687-021-00363-5 (PMC8433277; doi:10.1186/s41687-021-00363-5)
Supplement: Supplementary file 1 — Additional file 1. Representative LiverMultiScan Report. [file 41687_2021_363_MOESM1_ESM.pdf]

Additional file 1. Representative Liver*MultiScan™ Report**

*Reports shown to patients are of Liver*MultiScan™* Discover, the research platform of Liver*MultiScan™*

Liver MultiScan Discover

P15-03-112
release-LMS-Discover-3.1

This report was generated with research software and is not for clinical use

Patient name: **P15-03-112**	Referred by: **N/A**
Date of Birth: **N/A**	Series date: **28-Feb-2017**
Patient ID: **O3T-P15-03-1112**	Report date: **01-Mar-2017**
Scanner: **SIEMENS**	Scanning centre: **John Radcliffe Hospital**
Software version: **syngo MR B17**	

A

Statistics Summary

Region 1

Fat: **2.7 %**	Normal range: <5.6% [1]	**LIF 0.7**
Iron: **1.7 mg/g dry weight liver**	Normal range: <1.8mg/g [2]	
cT1: **750.4 ms**	Normal range: 645ms - 822ms [3]	

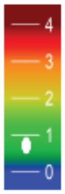

C

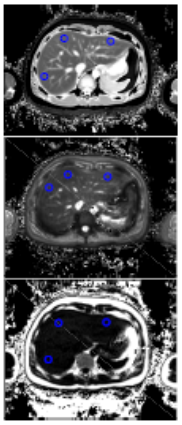

B

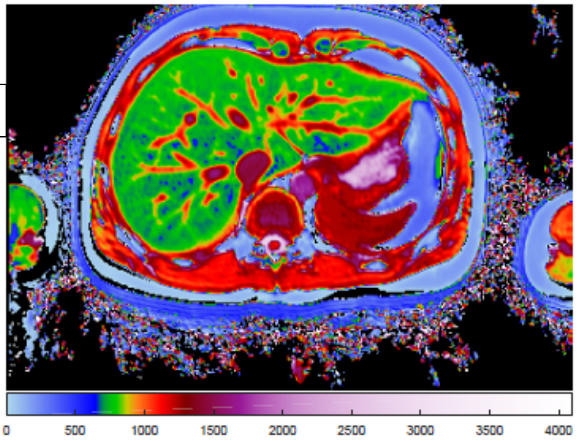

T1 image quality:	T2* image quality:
Fat image quality:	Additional comments:

[1] Szczepaniak et al, Am J Physiol Endocrinol Metab. 2005; 288(2):E462-8 Magnetic resonance spectroscopy to measure hepatic triglyceride content: prevalence of hepatic steatosis in the general population

[2] Nuttall et al, Ann Clin Lab Sci. 2003;33(4):443-50 Reference Limits for Copper and Iron in Liver Biopsies

[3] Banerjee et al, J Hepatol. 2014; 60(1): 6977 Multiparametric magnetic resonance for the non-invasive diagnosis of liver disease

Representative report for Liver*MultiScan™* Discover. At **(A)** there is the statistics summary for fat, iron and cT1 with accompanying LIF (*Liver Inflammation Fibrosis*) score. **(B)** shows the acquired image coloured to cT1 scoring with areas of green and blue representing lower cT1 scoring, and yellow and red higher. **(C)** is the raw unprocessed images acquired.

Advanced Analysis
Slice 1

cT1 values within the liver :

Mode: **737 ms**

Median: **766.59 ms**

Portion of liver with LIF [2 - 4] : **19 %**

Mean: **809.7 ms**

Interquartile range: **[731.17 - 839.72] ms**

A

C

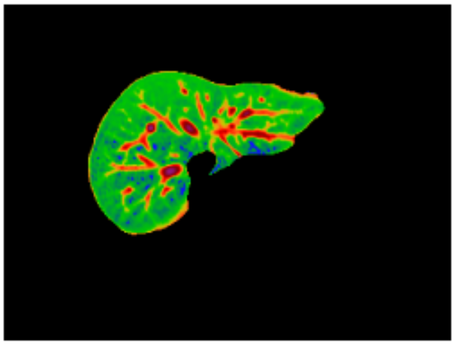

Segmented liver

B

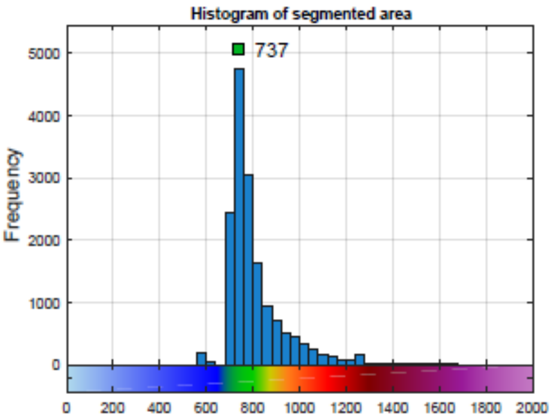

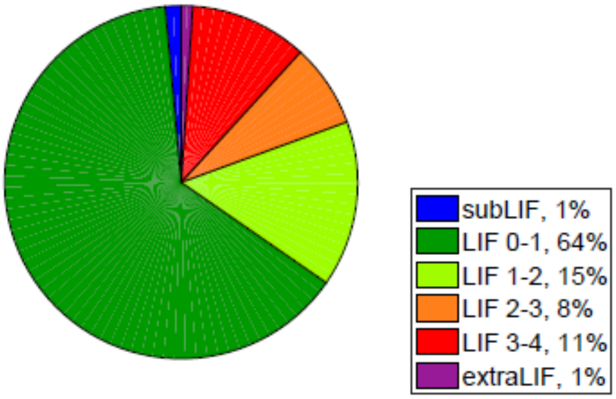

Representation of LIF classes within the liver

Representative report for Liver*MultiScan™* Discover advanced analysis. At **(A)** there is the image of the segmented liver with **(B)** showing how the cT1 scores map to LIF classes, showing overall spread of inflammation and fibrosis. **(C)** is a histogram showing the spread of cT1 scoring over the slice in question.
