# Supplementary material for: Patient understanding and experience of non-invasive imaging diagnostic techniques and the liver patient pathway
Source: J Patient Rep Outcomes. 2021 Sep 10;5:89. doi: 10.1186/s41687-021-00363-5 (PMC8433277; doi:10.1186/s41687-021-00363-5)
Supplement: Supplementary file 2 — Additional file 2. Representative Report of Echosens FibroScan® Report Card [file 41687_2021_363_MOESM2_ESM.pdf]

Additional file 2. Representative Report of Echosens Fibroscan® Report Card

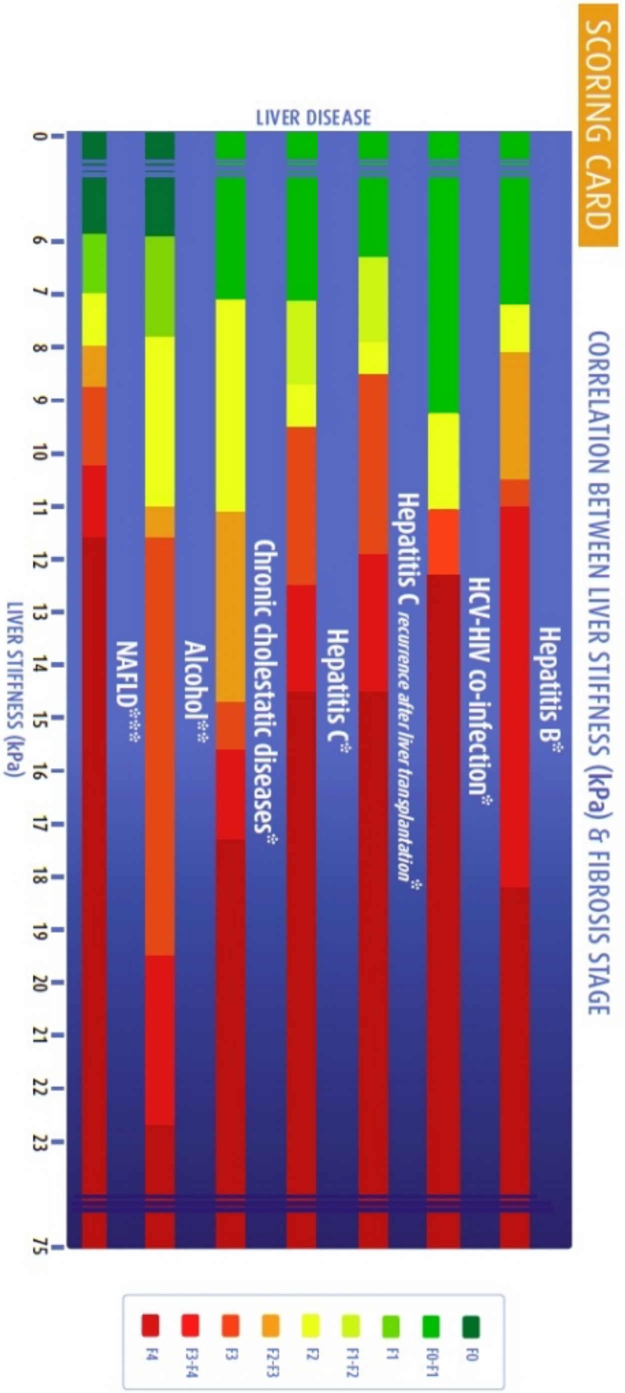

The FibroScan® Report Card uses colouring to demonstrate how it's output, Liver Stiffness (kPa) relates to fibrosis staging (F0 -F4) over different aetiologies.
