# Supplementary material for: Patient understanding and experience of non-invasive imaging diagnostic techniques and the liver patient pathway
Source: J Patient Rep Outcomes. 2021 Sep 10;5:89. doi: 10.1186/s41687-021-00363-5 (PMC8433277; doi:10.1186/s41687-021-00363-5)
Supplement: Supplementary file 3 — Additional file 3. Patient Group Designed Questionnaire. [file 41687_2021_363_MOESM3_ESM.pdf]

Additional file 3. Patient Group Designed Questionnaire

HEP C POSITIVE

Liver4Life

Perspectum Liver*MultiScan* Questionnaire

The following questions have been developed in collaboration with Liver4Life and Hep C Positive. This short questionnaire is designed as the basis for a focussed interview for patients who are undergoing a LiverMultiScan to better understand what kind of information they would like to receive during a LiverMultiScan test.

Even if patients receive bad news following the Scan, Perspectum are keen that the patient should feel confident with the service and next steps, to help alleviate any fears and anxieties.

These questions, designed to probe patient thoughts and understanding of LiverMultiScan, will be answered verbally during a semi-structured interview which will be recorded.

Questions 1-7 are to be completed before the scans.

1	**Please indicate which condition you have experience of either as a patient or carer** *(Please select)*	☐ Alcohol related liver condition<br>☐ Fatty liver or NAFLD<br>☐ Hepatitis B<br>☐ Hepatitis C<br>☐ Autoimmune hepatitis<br>☐ Primary Biliary Cholangitis<br>☐ Primary Sclerosing Cholangitis<br>☐ Gilberts Syndrome<br>☐ Unknown<br>☐ Other (please state) ____________
2	What is your sex? *(Please select)*	☐ Male<br>☐ Female<br>☐ Neither
3	Select your age range: *(Please select)*	☐ 16-25<br>☐ 26-35<br>☐ 36-45<br>☐ 46-55<br>☐ 56 or over

4	Please rate your understanding of your liver condition by circling a number<br>*1 = Poor understanding 10 = Great understanding*<br>1 | 2 | 3 | 4 | 5 | 6 | 7 | 8 | 9 | 10	
5	Would you like to receive information on the Liver*MultiScan* procedure before your appointment?	☐ Yes<br>☐ No
6	Please tell us where you currently get information about your liver condition:	☐ GP<br>☐ Consultant/Specialist nurse<br>☐ Local support group<br>☐ Social media<br>☐ Website *(please list most visited below)*<br>________________<br>☐ Other<br>________________
7	PRIOR TO the scan, please rate your understanding of the health state of your liver (ie level of damage/fibrosis/cirrhosis) by circling a number<br>*1 = Poor understanding 10 = Excellent understanding*<br>1 | 2 | 3 | 4 | 5 | 6 | 7 | 8 | 9 | 10	
	Questions 8 to 14 are to completed after the scans.	
8	AFTER the scan, please rate your understanding of the health state of your liver (ie level of damage/fibrosis/cirrhosis) by circling a number<br>*1 = Poor understanding 10 = Excellent understanding*<br>1 | 2 | 3 | 4 | 5 | 6 | 7 | 8 | 9 | 10	

9	How much did the explanation you received improve your understanding of the results of your scan?<br>*1 = Poor 10 = Excellent*<br>1 | 2 | 3 | 4 | 5 | 6 | 7 | 8 | 9 | 10<br>Would more detail or a different method of explaining the results improve your understanding?<br>Yes/no<br><br>If yes, please provide comments:									

10	How would you prefer to receive the information produced by the Liver*MultiScan*?	☐ Written information<br>☐ Written information including a 'liver score' and pictures<br>☐ Pictures only<br>☐ Face-to-face from specialist<br>☐ Online
11	Do you believe that other patients could benefit from receiving a Liver*MultiScan*?	☐ Yes<br>☐ No
	Based on your experience today, would you recommend the Liver*MultiScan* to other people with liver conditions?<br>*1 = Definitely would not recommend* *10 = Definitely will recommend*<br>1 | 2 | 3 | 4 | 5 | 6 | 7 | 8 | 9 | 10	
12	Would you be happy to participate in studies similar to this in the future?	☐ Yes<br>☐ No

13	**Preference of Liver*MultiScan* to ultrasound (FibroScan)**<br>Which scan did you feel was most useful?<br>Which scan did you find least tolerable, and why?<br>Overall which scan would you prefer to have as part of your treatment in future?	
14	**Please provide any additional comments and feedback**<br>**Prompts for researcher:**<br>(for example, tell us what your experience of the procedure was like?<br>How did you feel during the scans?<br>Did you feel well cared for by the staff?<br>Should there the opportunity/facility to take someone with you for the appointment?)	
