# Supplementary material for: Patient understanding and experience of non-invasive imaging diagnostic techniques and the liver patient pathway
Source: J Patient Rep Outcomes. 2021 Sep 10;5:89. doi: 10.1186/s41687-021-00363-5 (PMC8433277; doi:10.1186/s41687-021-00363-5)
Supplement: Supplementary file 4 — Additional file 4. GRIPP2 short form. [file 41687_2021_363_MOESM4_ESM.pdf]

GRIPP2 short form

**1: Aim**	This study aimed to better understand the experience of patients with a diagnosis of chronic liver disease. Specifically, we were interested in exploring the effects of non-invasive diagnostic tests for liver disease on patients' experiences in comparison to the standard of care that often utilizes biopsies to diagnose liver disease.
**2: Methods**	Patients and patient advocacy groups were involved in the design and implementation of the study, and analysis of the transcripts.
**3: Study results**	This study revealed that non-invasive diagnostic modalities are preferred over invasive tests for the diagnosis of liver disease. Involvement with patient groups was frequently associated with a better patient experience. Additional factors contributing to a better experience were test results with a visual component related to the degree of liver function and health professionals willing to take to explain the disease and the results of tests.
**4: Discussion and conclusions**	Having a patient group from an established charity working with liver disease was crucial to identify which issues were important for patients diagnosed with liver disease. In addition, the involvement of patients and patient groups greatly contributed to successful recruitment.
**5: Reflections/critical perspecive**	Although we were able to learn what aspects of care mattered most to patients and caregivers of patients with liver disease we were not able to determine if the addition of novel non-invasive diagnostic tests will affect clinical or patient reported outcomes. It is often hard to combine in a single study already established clinical outcomes with patient-centered and patient-reported outcomes. The increase in complexity and cost of such studies pose challenges related to funding and significance of the findings for all stakeholders. It would be advisable and ideal to have patient reported outcomes as standard endpoints in all studies evaluating new interventions or therapies that affect patients with liver disease.
